# Parent-Reported Communication Abilities of Children with Sotos Syndrome: Evidence from the Children’s Communication Checklist-2

**DOI:** 10.1007/s10803-018-3842-0

**Published:** 2018-12-08

**Authors:** Chloe Lane, Jo Van Herwegen, Megan Freeth

**Affiliations:** 1 Department of Psychology, University of Sheffield, Sheffield, S1 2LT UK; 2 Department of Psychology, Kingston University London, Kingston upon Thames, KT1 2EE UK

**Keywords:** Sotos syndrome, Williams syndrome, Communication, Language, Pragmatics

## Abstract

Sotos syndrome is a congenital overgrowth syndrome associated with intellectual disability. This study investigated communicative abilities of children with Sotos syndrome (n = 31), using the Children’s Communication Checklist, second edition. A cross-syndrome approach was used to establish the specificity of these abilities. Children with Williams syndrome (n = 34) were used as a comparison group. In both groups, the majority of participants had communicative impairment. Children with Sotos syndrome had an uneven pragmatic language profile and greater impairment with social relations, compared with restricted interests. Overall, children with Sotos syndrome had difficulties with both language structure and pragmatic language and a specific profile of relative difficulty with using nonverbal communication, using context-appropriate language and understanding peer relationships.

Sotos syndrome is a congenital overgrowth syndrome with an estimated incidence of approximately 1 in 14,000 (Tatton-Brown and Rahman 2004). The cardinal features of the syndrome are overgrowth with advanced bone age, macrocephaly, characteristic facial features and intellectual disability (Tatton-Brown et al. 2005). The syndrome is caused by abnormality of the NSD1 (nuclear receptor binding SET domain protein 1) gene, which is located on chromosome 5 (Kurotaki et al. 2002). Until recently, the cognitive and behavioural phenotype associated with Sotos syndrome was considerably under-researched and much of the literature was based on small samples (Lane et al. 2016). However, recent research has investigated the phenotype using larger and more representative samples and this has established that Sotos syndrome is associated with a high prevalence of behavioural symptomatology associated with autism spectrum disorder (ASD), as well as a cognitive profile of relative strength with verbal ability but relative weakness with nonverbal reasoning ability (Lane et al. 2017, 2018; Sheth et al. 2015). The majority of individuals with Sotos syndrome have mild to moderate intellectual disability and in a recent cohort study, the range of reported general conceptual ability (GCA) scores, as assessed by the British Ability Scales 3 (BAS3), was 37–101, with a mean of 60.75 (Lane et al. 2018).

Cross-syndrome comparisons are useful for assessing similarities and differences between phenotypes associated with distinct syndromes and can lead to identification of unique characteristic features of syndromes. These comparisons enable the specificity of strengths and difficulties associated with a particular syndrome to be established and can inform understanding of specific factors to target for intervention. Williams syndrome (WS) is a congenital syndrome with a similar incidence to that of Sotos syndrome (Strømme et al. 2002). WS provides a useful comparison for Sotos syndrome for a number of reasons. Specifically, both syndromes are associated with similar overall IQ (mean IQ of approximately 60) and a profile of better verbal ability compared with nonverbal reasoning ability (Lane et al. 2018; Martens et al. 2008; Mervis et al. 2000; Udwin and Yule 1991). However, despite better verbal ability compared with nonverbal reasoning ability, WS is associated with delayed and atypical language development (Brock 2007). In addition, social difficulties have been reported in both syndromes, although the exact nature of these difficulties has not yet been established for individuals with Sotos syndrome (Davies et al. 1998; Doyle et al. 2004; Lane et al. 2017; Sheth et al. 2015). Thus, comparison of the language and communication abilities of children with these syndromes will enable subtle differences in the phenotypes to be identified, irrespective of general cognitive ability and social difficulties.

Language and communication abilities are fundamental for human interaction. Effective communication can facilitate learning and enable individuals to share information and ideas. In order to structure language correctly, it is necessary to have an understanding of the rules governing language structure (e.g. the ability to construct coherent sentences in which words are used in the correct order). In addition, effective communication requires the ability to understand how to use language appropriately, or pragmatic language (e.g. using context-appropriate language and understanding the communicative partners’ experience of the interaction). Language structure ability is not necessarily related to pragmatic language ability. For example, developmental language disorder (DLD) is associated with relative difficulty with structural aspects of language, compared with functional pragmatic language, whereas ASD is associated with relative difficulty with pragmatic aspects of language, compared with language structure (Geurts and Embrechts 2008; Norbury et al. 2004). In addition, children with Down syndrome typically display greater difficulty with language structure skills, compared with pragmatic language skills, whilst children with WS have greater difficulty with pragmatic language (Hoffmann et al. 2013; Smith et al. 2017). This demonstrates the specificity of language profiles associated with different conditions.

Several studies have reported communication impairment and language delays in Sotos syndrome (Lane et al. 2016). Finegan et al. (1994) conducted the most comprehensive study of language skills in individuals with Sotos syndrome to date and found that language ability was consistent with overall intellectual ability. However, this study only focused on the discrepancy between verbal comprehension and expressive language and therefore did not investigate specific communication abilities, such as pragmatic language and language structure. In relation to language ability in Sotos syndrome, the majority of studies have used small samples and the prevalence and nature of communicative impairments has not been explored in detail (Lane et al. 2016). It is therefore important to establish whether individuals with Sotos syndrome display a consistent and characteristic profile of communication impairment in relation to structural and pragmatic aspects of language and the extent to which these linguistic abilities are related.

The Children’s Communication Checklist, second edition (CCC-2) (Bishop 2003) is designed to assess communicative abilities in children and is a valid measure for differentiating between individuals with distinct communicative impairments, including those with developmental disabilities (Norbury et al. 2004; Volden and Phillips 2010). Laws and Bishop (2004) used a cross-syndrome approach to investigate differences in pragmatic language impairment and social deficits, as assessed by the Children’s Communication Checklist (Bishop 1998), in children and young adults with WS, Down syndrome and specific language impairment (SLI). The findings from this study demonstrated that participants with WS had significant pragmatic language impairment and had particular difficulty with inappropriate initiation of conversation and stereotyped language, compared with both the Down syndrome and SLI participants. This demonstrates that exploration of pragmatic language profiles is useful for differentiating between syndromes and for identifying specific areas of relative difficulty associated with distinct conditions. In addition, participants with WS could be differentiated from the other groups on the basis of experiencing a lack of normal inhibition with strangers. This has been widely reported as a characteristic feature of the social phenotype of WS and is a valuable insight into the social difficulties associated with WS (Doyle et al. 2004; Riby et al. 2014). Therefore, the CCC-2 is an effective measure for characterising and differentiating between the social and communicative phenotypes associated with different conditions and for identifying syndrome-specific difficulties.

To date, language structure skills and pragmatic language ability have not been explored within the Sotos syndrome population and understanding of the communicative abilities of individuals within this population is limited. It is important to identify the prevalence and profile of language and communication difficulties within the Sotos syndrome population as impairments may have social and educational implications. For example, language skills are critical for social interaction so may impact peer relationships and identification of language and communication difficulties will enable appropriate services and provisions to be identified for individuals with Sotos syndrome. In addition, comparison of the strengths and difficulties of children with Sotos syndrome with those of children with WS will enable the specificity of the language and communicative profile associated with Sotos syndrome to be established. Once specific impairments have been established, these can be explored further in order to identify factors which may underlie these impairments. Ultimately, this approach will enable optimal strategies and interventions to be devised to address specific impairments.

The primary aim of this study was to investigate the communication abilities of children with Sotos syndrome and to identify relative communicative strengths and difficulties associated with this syndrome, as assessed by the CCC-2. It was hypothesised that children with Sotos syndrome would have significant communicative difficulties. A secondary aim of this study was to compare the communicative abilities of children with Sotos syndrome with those of a matched group of children with WS in order to determine whether the communicative profiles are syndrome-specific. This is the first cross-syndrome comparison of Sotos syndrome and WS.

## Method

### Participants

The sample comprised 31 children with Sotos syndrome (17 males, 14 females) and 34 children with WS (18 males, 16 females), matched on chronological age and sex. Mean age of the Sotos syndrome participants was 9.12 years (SD = 3.81; range = 4–16.42 years) and mean age of the WS participants was 9.4 years (SD = 3.33; range = 4–16.75 years). The parent/caregiver of each child completed the CCC-2. Families of children with Sotos syndrome were recruited via the Child Growth Foundation (CGF; a UK charity that supports families of individuals affected by growth disorders) and advertisements on a Sotos syndrome support group on social media. Families of children with WS were recruited via the Williams Syndrome Foundation (a UK charity that supports families of individuals with WS).

### Measures

The CCC-2 is a standardised 70-item questionnaire designed to assess communicative abilities in children (4–16 years 11 months) and can be used to identify children with significant communicative problems. Age-appropriate norms are provided. Items are coded on a Likert scale to determine the frequency of communicative difficulties and communicative strengths (0 = less than once a week or never to 3 = several times a day or always). The CCC-2 has 10 subscales which assess: (A) speech; (B) syntax; (C) semantics; (D) coherence; (E) inappropriate initiation; (F) stereotyped language; (G) use of context; (H) nonverbal communication; (I) social relations; (J) interests. Each of the subscales has 7 items; 5 relate to communicative difficulties and 2 relate to communicative strengths. A General Communication Composite (GCC) score provides an indication of the communicative ability of a child and is calculated as the sum of scaled scores for subscales A–H. Based on the standardisation sample, a GCC score of 54 is equivalent to the 10th percentile and GCC scores < 28 are equivalent to < 1st percentile. In addition, a language structure score (sum of scaled scores for subscales A, B, C and D) and a pragmatic language score (sum of scaled scores for subscales E, F, G and H) can be calculated in order to directly compare language structure skills and pragmatic language skills. For both language structure and pragmatic language, scores > 24 indicate typical functioning, scores of 17–24 indicate borderline functioning and scores < 17 indicate impaired functioning. The CCC-2 has been found to be a valid measure for differentiating between individuals with typical communicative ability and those with significant communicative impairments and within the CCC-2 standardisation sample, internal consistency of the subscales ranged from 0.65 to 0.80 (Bishop 2003).

## Results

### Overall Communication Ability

Communication ability was assessed on the basis of GCC scores, with GCC scores < 55 (≤ 10th percentile) indicating that a child has significant communicative difficulties. For participants with Sotos syndrome, GCC scores ranged from 1 to 57 (< 1st percentile–13th percentile), demonstrating that the majority of children with Sotos syndrome had communicative difficulties of varying severity, with only one participant scoring in the normal range. GCC scores for participants with WS ranged from 4 to 60 (< 1st percentile–15th percentile), indicating that the majority of children with WS had communicative difficulties and once again, only one participant scored in the normal range. An independent samples *t*-test was used to compare overall communicative ability of children with Sotos syndrome and children with WS. The analysis revealed no significant difference between GCC scores for the Sotos syndrome participants (M = 27.55, SD = 14.81) and GCC scores for the WS participants (M = 26.82, SD = 10.60); *t*(63) = 0.23, *p* = .820, *d* = 0.06, indicating that both syndromes are associated with a similar level of overall communicative ability.

### Language Structure and Pragmatic Language

In order to determine whether children with Sotos syndrome had an uneven profile of greater difficulty with either language structure or pragmatic language, scores for these abilities were compared. The Sotos syndrome profile was also compared to the WS profile to establish any syndrome-specific differences. Language structure scores were calculated as the sum of scaled scores for subscales A, B, C and D for participants with Sotos syndrome (M = 12.68, SD = 8.38) and participants with WS (M = 13.97, SD = 7.11). Pragmatic language scores were calculated as the sum of scaled scores for subscales E, F, G and H for participants with Sotos syndrome (M = 14.87, SD = 8.16) and participants with WS (M = 12.85, SD = 5.85). A 2 × 2 (Group: Sotos/WS x Language skill: structure/pragmatic) mixed measures ANOVA revealed no main effect of skill; *F*(1, 63) = 0.34, *p* = .564, *ηρ²* = .005, indicating no discrepancy between language structure skills and pragmatic language skills overall within this cohort. There was no effect of group; *F*(1, 63) = 0.05, *p* = .820, *ηρ²* = .001 and no skill x group interaction; *F*(1, 63) = 3.18, *p* = .079, *ηρ²* = .048. This suggests that both children with Sotos syndrome and children with WS had a similar degree of impairment with language structure skills and pragmatic language skills.

A Chi square test of independence was used to assess the association between group (Sotos/WS) and category of language ability (impaired/borderline/typical). There was no significant association between group and language structure ability category, *X*^2^(2, *N* = 65) = 0.44, *p* = .801, and no significant association between group and pragmatic language ability category, *X*^2^(2, *N* = 65) = 3.40, *p* = .183. This indicates that, for both Sotos syndrome and WS, a similar proportion of individuals had impaired, borderline and typical language structure and pragmatic language abilities (see Table 1).

**Table 1:** Proportion of participants in the impaired functioning (scores < 17), borderline functioning (scores 17–24) and typical functioning (scores > 24) categories for language structure scores and pragmatic language scores

	Impaired functioning (%)	Borderline functioning (%)	Typical functioning (%)
Language structure			
Sotos	61.3	29.0	9.7
WS	67.6	26.5	5.9
Pragmatic language			
Sotos	64.5	19.4	16.1
WS	76.5	20.6	2.9

Although children with Sotos syndrome had similar overall difficulty with both language structure and pragmatic language abilities, there was significant variability in level of functioning of these abilities within each group. In order to investigate this variability, multiple regression was used to establish the extent to which variance in pragmatic language ability is explained by language structure skills, age and sex. The regression equation was significant; *R*^*2*^ = 0.64, *F*(3, 27) = 16.27, *p* < .001, and inspection of the beta weights revealed that both language structure skills (*β* = 0.61, *p* < .001) and age (*β* = − 0.53, *p* < .001) were significant predictors of pragmatic language ability. The same analysis was used to assess variance in pragmatic language ability for participants with WS. The regression equation was significant; *R*^*2*^ = 0.25, *F*(3, 30) = 3.33, *p* = .033, and inspection of the beta weights revealed that both language structure skills (*β* = 0.39, *p* = .023) and age (*β* = − 0.37, *p* = .032) were significant predictors of pragmatic language ability. This suggests that language structure skills and age are significant predictors of pragmatic language ability for both children with Sotos syndrome and children with WS. Although the predictors were the same in both groups, language structure skills and age explained more variance in pragmatic language ability for children with Sotos syndrome (64%) than children with WS (25%). This indicates that other factors may account for variability in pragmatic language ability within the WS population.

### Language Structure Subscale Profile

In order to determine whether children with Sotos syndrome had particular difficulty with specific aspects of language structure, a repeated measures ANOVA was used to compare scaled scores for the four language structure subscales: (A) speech (e.g. pronunciation), (B) syntax (e.g. use of pronouns), (C) semantics (e.g. differentiation of words which sound similar) and (D) coherence (e.g. description of a sequence of events). A scaled subscale score ≤ 5 is indicative of significant communicative problems. The analysis identified no effect of subscale; *F*(3, 90) = 2.16, *p* = .099, *ηρ²* = .067, indicating that children with Sotos syndrome had similar ability in all four language structure skills assessed by the CCC-2.

**Fig. 1:**
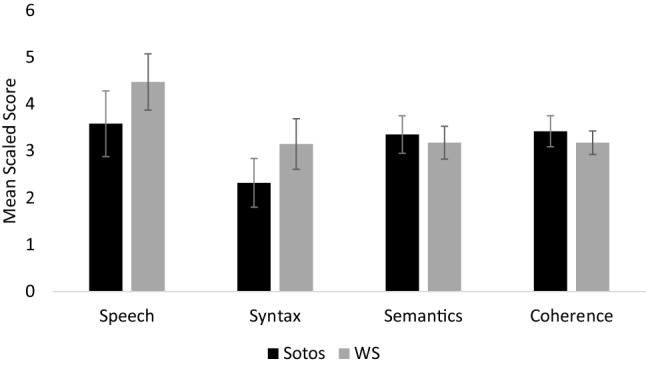
Mean scaled scores for the four language structure subscales (subscales A–D) for the Sotos and Williams syndrome (WS) groups. Error bars show +/− 1 standard error

The language structure skills of children with Sotos syndrome were compared with those of children with WS in order to investigate syndrome-specific differences in these abilities. A 2 × 4 (Group: Sotos/WS x Language structure subscales: A, B, C, D) mixed measures ANOVA was used to compare scaled scores for the four language structure subscales. The analysis identified a significant main effect of subscale; *F*(3, 189) = 3.61, *p* = .014, *ηρ²* = .054 but no significant subscale x group interaction; *F*(3, 189) = 1.23, *p* = .302, *ηρ²* = .019 and no significant effect of group; *F*(1, 63) = 0.45, *p* = .504, *ηρ²* = .007. As there was no significant subscale x group interaction, this suggests that children with these syndromes had similar difficulty with the language structure skills assessed by the CCC-2. Figure 1 shows mean scaled scores for the language structure subscales.

### Pragmatic Language Subscale Profile

In order to determine whether children with Sotos syndrome had particular difficulty with specific aspects of pragmatic language, a repeated measures ANOVA was used to compare scaled scores for the four pragmatic language subscales: (E) inappropriate initiation (e.g. telling people things they already know), (F) stereotyped language (e.g. repeating back things other people have said), (G) use of context (e.g. consistency of communication skills across different situations) and (H) nonverbal communication (e.g. use of facial expressions and eye contact). The analysis identified a significant effect of subscale; *F*(3, 90) = 11.32, *p* < .001, *ηρ²* = .274, indicating that children with Sotos syndrome had an uneven profile of pragmatic language skills. Post-hoc paired samples *t*-tests (using a Bonferroni correction, *p* < .008 required for significance) were used to compare scaled scores for the four pragmatic language subscales. The significant comparisons were stereotyped language > use of context, *t*(30) = 5.67, *p* < .001, *d* = 1.02; stereotyped language > nonverbal communication, *t*(30) = 4.68, *p* < .001, *d* = 0.84; inappropriate initiation > use of context, *t*(30) = 4.60, *p* < .001, *d* = 0.83. There was a trend for inappropriate initiation > nonverbal communication, *t*(30) = 2.76, *p* = .01, *d* = 0.49. This suggests that, in terms of the pragmatic language profile, children with Sotos syndrome have greater difficulty with use of context and nonverbal communication, whereas inappropriate initiation and stereotyped language are less problematic.

The pragmatic language abilities of children with Sotos syndrome were compared with those of children with WS in order to determine the specificity of the Sotos syndrome pragmatic language profile. A 2 × 4 (Group: Sotos/WS x Pragmatic language subscales: E, F, G, H) mixed measures ANOVA was used to compare scores for the four pragmatic language subscales. The analysis identified no significant effect of group; *F*(1, 63) = 1.33, *p* = .253, *ηρ²* = .021. There was a significant main effect of subscale; *F*(3, 189) = 17.58, *p* < .001, *ηρ²* = .218, and a significant subscale x group interaction; *F*(3, 189) = 4.86, *p* = .003, *ηρ²* = .072, indicating that both groups had uneven pragmatic language profiles but the nature of these profiles differed.

**Fig. 2:**
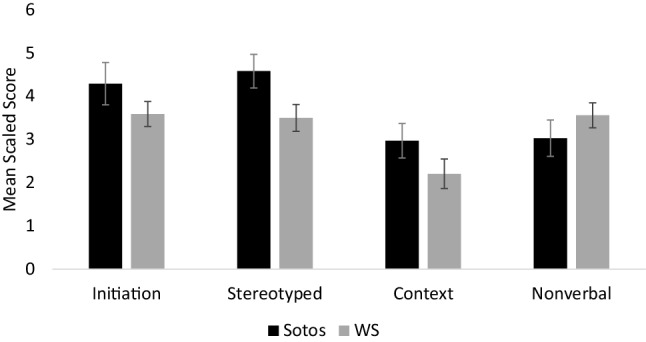
Mean scaled scores for the four pragmatic language subscales (subscales E–H) for the Sotos and Williams syndrome (WS) groups. Error bars show +/− 1 standard error

As there was a significant subscale x group interaction, post-hoc comparisons (using a Bonferroni correction *p* < .013 required for significance) were used to compare scores for the four pragmatic language subscales between the groups. The comparisons revealed a trend for the Sotos syndrome participants to have better stereotyped language scores, compared with the WS participants, *t*(63) = 2.19, *p* = .032, *d* = 0.55. There were no significant differences between groups for inappropriate initiation scores, *t*(63) = 1.27, *p* = .210, *d* = 0.32; use of context scores, *t*(63) = 1.46, *p* = .148, *d* = 0.37; or nonverbal communication scores, *t*(63) = -1.05, *p* = .298, *d* = 0.26. This suggests that the Sotos syndrome pragmatic language profile is characterised by less difficulty with stereotyped language. Figure 2 shows mean scaled scores for the pragmatic language subscales.

### Autism Subscale Profile

The CCC-2 has two subscales which assess behaviours typically associated with ASD; social relations (e.g. interaction with other children) and interests (e.g. preference for the same favourite activity). In order to determine whether children with Sotos syndrome had relative differences in these behaviours, a paired samples *t*-test was used to compare these subscale scores. The analysis revealed a significant difference between the social relations subscale and the interests subscale, *t*(30) = − 5.99, *p* < .001, *d* = 1.08, indicating that children with Sotos syndrome had greater difficulty with social relations, compared with restricted interests. In order to establish whether this profile of behaviours was syndrome-specific, a 2 × 2 (Group: Sotos/WS x ASD subscales: I, J) mixed measures ANOVA was used to compare scaled scores for the ASD subscales between participants with Sotos syndrome and participants with WS. The analysis revealed a significant main effect of subscale; *F*(1, 63) = 27.74, *p* < .001, *ηρ²* = .306 and a significant subscale x group interaction; *F*(1, 63) = 8.79, *p* = .004, *ηρ²* = .122, indicating that both groups had uneven ASD subscale profiles but the nature of these profiles differed. There was no effect of group; *F*(1, 63) = 0.04, *p* = .835, *ηρ²* = .001.

**Fig. 3:**
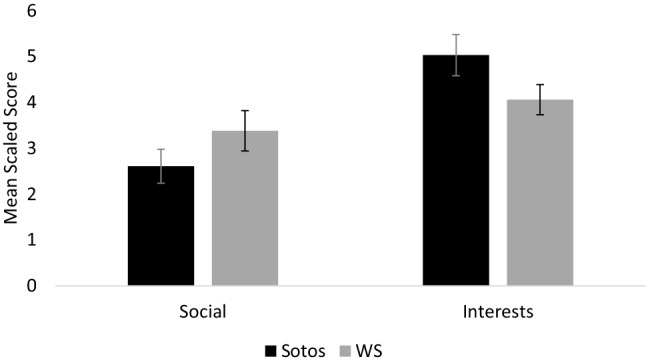
Mean scaled scores for the two autism subscales (subscales I–J) for the Sotos and Williams syndrome (WS) groups. Error bars show +/− 1 standard error

As there was a significant subscale x group interaction, post-hoc comparisons (using a Bonferroni correction *p* < .025 required for significance) were used to compare scores for the two ASD subscales within the Sotos syndrome and WS participants. As reported above, participants with Sotos syndrome had significantly greater difficulty with social relations, compared with restricted interests. However, the same analysis with participants with WS revealed no significant difference between the social relations subscale and the interests subscale, *t*(33) = − 1.60, *p* = .120, *d* = 0.27. Overall, this suggests that children with Sotos syndrome tend to have less difficulty with restricted interests compared with social relations, and this profile was specific to the Sotos syndrome participants. Figure 3 shows mean scaled scores for these subscales.

## Discussion

The primary aim of this study was to investigate communicative abilities of children with Sotos syndrome, using the CCC-2. A cross-syndrome approach was used to compare communicative abilities of children with Sotos syndrome with a matched group of children with WS in order to investigate the specificity of the communicative profile associated with Sotos syndrome. The findings indicate that both syndromes are associated with communicative impairment and a similar level of overall communicative ability. There were no relative differences between overall language structure skills and pragmatic language skills, indicating that children with Sotos syndrome have similar difficulty with both of these linguistic abilities. This was also observed for children with WS. Furthermore, language structure skills and age were significant predictors of pragmatic language ability for both syndromes but accounted for greater variation in pragmatic language ability for the Sotos syndrome participants. Within the Sotos syndrome group, children had an uneven pragmatic language profile and ASD profile but similar difficulty with all language structure abilities assessed by the CCC-2. Specifically, the pragmatic language profile was characterised by greater difficulty with use of context and nonverbal communication but relatively less difficulty with inappropriate initiation and stereotyped language. The ASD profile was characterised by greater difficulty with social relations, compared with restricted interests. Both the pragmatic language profile and ASD profile were specific to the Sotos syndrome participants.

The present study included a sample of 31 children with Sotos syndrome and this is the largest study to date to explore communicative abilities within this population. Overall, children with Sotos syndrome had communicative difficulties, compared with typically developing peers of the same age, as evidenced by low GCC scores. Only one participant with Sotos syndrome had a GCC score > 10th percentile. Furthermore, there was no significant difference between GCC scores for children with Sotos syndrome and children with WS, indicating that these populations are associated with a similar degree of overall communicative ability. However, it is important to note that there was considerable variability in communicative ability within each group. Previous research has established that both Sotos syndrome and WS are associated with relative strength in verbal ability, compared with nonverbal reasoning ability, as assessed by standardised cognitive assessments (Lane et al. 2018; Udwin and Yule 1991). However, despite having relative strength with verbal ability, individuals with both of these syndromes have significant difficulty with communication skills, relative to typically developing peers. It is important to note that this is based on interpretation of communication skills in relation to the CCC-2 standardisation sample and that the present study did not include a typically developing control group. In addition, IQ and adaptive functioning were not assessed within the present study so communicative abilities have not been compared with overall intellectual ability for participants with Sotos syndrome. Overall, the findings demonstrate the need for children with Sotos syndrome to receive support with the development of their language and communication skills, as the majority of children with Sotos syndrome have impaired communicative abilities.

Previous research indicates that different conditions are associated with uneven profiles of language structure and pragmatic language abilities (Geurts and Embrechts 2008; Hoffmann et al. 2013; Norbury et al. 2004; Smith et al. 2017). In the present study, comparison of these linguistic abilities for children with Sotos syndrome revealed no significant difference between overall language structure skills and pragmatic language skills, indicating that children with Sotos syndrome have similar difficulty with both understanding how to structure language and use of appropriate language. Children with WS also had no discrepancy between these abilities and there was no significant difference between the two groups in terms of overall ability for either of these linguistic skills. This indicates that the communicative abilities of children with Sotos syndrome are distinct to both DLD and ASD, which are both associated with relative difficulties with structural aspects of language and functional pragmatic language, respectively, and that children with Sotos syndrome will likely require support with the development of both aspects of language (Geurts and Embrechts 2008; Norbury et al. 2004). In addition, language structure skills and age were significant predictors of pragmatic language ability for both syndromes but accounted for greater variation in pragmatic language ability for children with Sotos syndrome. This suggests that, for children with Sotos syndrome, better understanding of the rules governing language structure is a significant predictor of better use of language. Thus, this demonstrates the importance of supporting children with Sotos syndrome with the development of structural language skills such as syntax, semantics, pronunciation, grammar and how to produce coherent speech.

Although previous research has reported that children with Sotos syndrome have communication impairment and language delays, the communicative profile had not been explored in detail (Finegan et al. 1994; Lane et al. 2016). In the present study, comparison of the individual CCC-2 subscales revealed that children with Sotos syndrome had similar difficulty with all aspects of language structure but had an uneven profile of pragmatic language abilities. Specifically, participants had greater impairment with use of context and nonverbal communication, compared with inappropriate initiation and stereotyped language. Relative difficulty with nonverbal communication is consistent with previous findings of relative difficulty with nonverbal cognitive ability within the Sotos syndrome population (Lane et al. 2018). However, difficulty with using context-appropriate language is a novel finding which has not previously been reported within the Sotos syndrome literature. In addition, the pragmatic language profiles differed between the syndromes. Specifically, children with Sotos syndrome had less impairment with stereotyped language, compared with children with WS, indicating that repetition of phrases and use of over-precise information is less problematic for children with Sotos syndrome. This supports findings from previous cross-syndrome comparisons which have demonstrated that children with WS have greater difficulty with stereotyped language, compared with children with Down syndrome and children with SLI (Laws and Bishop 2004). Previous research indicates that pragmatic language difficulties may be accounted for by factors such as impaired theory of mind (ToM), weak central coherence (WCC) and executive dysfunction (Martin and Mcdonald 2003). Therefore, as the findings from the present study indicate that children with Sotos syndrome have impaired pragmatic language skills, it will be important for future research to assess factors such as ToM, WCC and executive functions in children with Sotos syndrome in order to determine whether these are also impaired. This will provide insight into potential factors underlying pragmatic difficulties for individuals with Sotos syndrome.

Although children with Sotos syndrome had difficulty with both ASD subscales, participants had significantly greater impairment with social relations, compared with restricted interests. Difficulty with these behaviours is consistent with previous research indicating that Sotos syndrome is associated with increased prevalence of behaviours typically associated with ASD (Lane et al. 2017; Sheth et al. 2015). Within the CCC-2, the majority of items included in the social relations subscale assess behaviours related to peer relationships, such as hurting or upsetting other children without meaning to, being teased or bullied by other children and appearing anxious in the company of other children. Overall, children with Sotos syndrome had significant difficulty with this subscale, indicating that forming and maintaining peer relationships may be problematic. As children with Sotos syndrome are often noticeably taller than their peers, other children may mistake them as being older so this could account for some of the difficulties with peer relationships. Previous research indicates that individuals with WS often struggle with forming and maintaining friendships (Davies et al. 1998). Within the present study, children with Sotos syndrome were reported as experiencing similar impairment with social relations as children with WS, indicating that both syndromes are associated with significant difficulties with peer relationships. This has not previously been reported within the Sotos syndrome literature. It will be important for future research to specifically investigate peer relationships within the Sotos syndrome population and to determine the extent to which social interaction difficulties result in problems with peer relationships, as this may impact social development and wellbeing.

Due to the rarity of the syndrome, the present study used a parental questionnaire to assess communicative abilities, in order to obtain a relatively large sample of children with Sotos syndrome. The advantage of this methodology is that parents spend a lot of time with their children so are familiar with abnormal communicative abilities which may not be observed during a clinical test or observation. However, it will be important for future research to further understanding of the social and communicative abilities associated with Sotos syndrome by using paradigms during live social interactions, in order to directly assess these skills in children with the syndrome. For example, this approach has been used to investigate social attention in children with autism (Hanley et al. 2014). A benefit of this approach is that communicative abilities can be observed within social contexts and responses can be compared between participants.

The present study focused on children with Sotos syndrome but it will be important for future research to investigate language and communication in adults with Sotos syndrome. Previous research indicates that difficulties with social interaction, restricted interests and repetitive behaviours may be less severe in adulthood, compared with childhood, within the Sotos syndrome population (Lane et al. 2017). Language and communication impairments have been found to have a long-term impact on other aspects of an individual’s life, such as relationship formation (Whitehouse et al. 2009) and behavioural, social and emotional problems (Snowling et al. 2006, St Clair et al. 2011). Thus, it will be useful for future research to determine whether communication and language skills improve in adulthood and the long-term impact that these impairments may have on social interaction, social relationships and emotional and behavioural difficulties for adults with Sotos syndrome.

## Conclusion

In summary, the findings from the present study demonstrate that the majority of children with Sotos syndrome have communicative impairment, compared with typically developing peers and that children within this population struggle with both language structure and pragmatic language. Children with Sotos syndrome have an uneven pragmatic language profile characterised by relative difficulty with use of context and nonverbal communication, compared with inappropriate initiation and stereotyped language. This pragmatic language profile was specific to Sotos syndrome. Overall, the findings inform understanding of specific communicative difficulties that are common in children with Sotos syndrome and demonstrate the need for support with language and communication development within this population. In particular, children with Sotos syndrome may require additional support with understanding and using nonverbal communication and using context-appropriate language and may have difficulty with peer relationships.
